# Understanding the complex genetic architecture connecting rheumatoid arthritis, osteoporosis and inflammation: discovering causal pathways

**DOI:** 10.1093/hmg/ddac061

**Published:** 2022-03-29

**Authors:** Melody Kasher, Frances M K Williams, Maxim B Freidin, Ida Malkin, Stacey S Cherny, CHARGE Inflammation Working Group, Emelia Benjamin, Daniel I Chasman, Abbas Dehghan, Tarunveer Singh Ahluwalia, James Meigs, Russell Tracy, Behrooz Z Alizadeh, Symen Ligthart, Josh Bis, Gudny Eiriksdottir, Nathan Pankratz, Myron Gross, Alex Rainer, Harold Snieder, James G Wilson, Bruce M Psaty, Josee Dupuis, Bram Prins, Urmo Vaso, Maria Stathopoulou, Lude Franke, Terho Lehtimaki, Wolfgang Koenig, Yalda Jamshidi, Sophie Siest, Ali Abbasi, Andre G Uitterlinden, Mohammadreza Abdollahi, Renate Schnabel, Ursula M Schick, Ilja M Nolte, Aldi Kraja, Yi-Hsiang Hsu, Daniel S Tylee, Alyson Zwicker, Rudolf Uher, George Davey-Smith, Alanna C Morrison, Andrew Hicks, Cornelia M van Duijn, Cavin Ward-Caviness, Eric Boerwinkle, J Rotter, Ken Rice, Leslie Lange, Markus Perola, Eco de Geus, Andrew P Morris, Kari Matti Makela, David Stacey, Johan Eriksson, Tim M Frayling, Eline P Slagboom, Gregory Livshits

**Affiliations:** Human Population Biology Research Unit, Department of Anatomy and Anthropology, Sackler Faculty of Medicine, Tel Aviv University, Tel Aviv 6997801, Israel; Department of Twin Research and Genetic Epidemiology, School of Life Course Sciences, King’s College London, London WC2R 2LS, UK; Department of Twin Research and Genetic Epidemiology, School of Life Course Sciences, King’s College London, London WC2R 2LS, UK; Human Population Biology Research Unit, Department of Anatomy and Anthropology, Sackler Faculty of Medicine, Tel Aviv University, Tel Aviv 6997801, Israel; Human Population Biology Research Unit, Department of Anatomy and Anthropology, Sackler Faculty of Medicine, Tel Aviv University, Tel Aviv 6997801, Israel; Department of Epidemiology and Preventive Medicine, Sackler Faculty of Medicine, Tel Aviv University, Tel Aviv 6997801, Israel; Human Population Biology Research Unit, Department of Anatomy and Anthropology, Sackler Faculty of Medicine, Tel Aviv University, Tel Aviv 6997801, Israel; Department of Twin Research and Genetic Epidemiology, School of Life Course Sciences, King’s College London, London WC2R 2LS, UK; Adelson Medical School, Ariel University, Ariel 40700, Israel

## Abstract

Rheumatoid arthritis (RA) and osteoporosis (OP) are two comorbid complex inflammatory conditions with evidence of shared genetic background and causal relationships. We aimed to clarify the genetic architecture underlying RA and various OP phenotypes while additionally considering an inflammatory component, C-reactive protein (CRP). Genome-wide association study summary statistics were acquired from the GEnetic Factors for OSteoporosis Consortium, Cohorts for Heart and Aging Research Consortium and UK Biobank. Mendelian randomization (MR) was used to detect the presence of causal relationships. Colocalization analysis was performed to determine shared genetic variants between CRP and OP phenotypes. Analysis of pleiotropy between traits owing to shared causal single nucleotide polymorphisms (SNPs) was performed using PL eiotropic A nalysis under CO mposite null hypothesis (PLACO). MR analysis was suggestive of horizontal pleiotropy between RA and OP traits. RA was a significant causal risk factor for CRP (*β* = 0.027, 95% confidence interval = 0.016–0.038). There was no evidence of CRP→OP causal relationship, but horizontal pleiotropy was apparent. Colocalization established shared genomic regions between CRP and OP, including *GCKR* and *SERPINA1* genes. Pleiotropy arising from shared causal SNPs revealed through the colocalization analysis was all confirmed by PLACO. These genes were found to be involved in the same molecular function ‘protein binding’ (GO:0005515) associated with RA, OP and CRP. We identified three major components explaining the epidemiological relationship among RA, OP and inflammation: (1) Pleiotropy explains a portion of the shared genetic relationship between RA and OP, albeit polygenically; (2) RA contributes to CRP elevation and (3) CRP, which is influenced by RA, demonstrated pleiotropy with OP.

## Introduction

Rheumatoid arthritis (RA) is a debilitating autoimmune disease with a prevalence of approximately 1% in the adult population (1), where females are at least twice as likely to be affected than males. The clinical appearance of RA typically manifests symmetrically in the smaller synovial joints of the hand and feet and may also affect large joints (2). Clinical implications of RA also extend to a variety of tissues, including lungs and eyes, and lead to bone and cartilage deterioration including osteoporosis (OP) resulting in a high burden of disease (2). There is a considerable disability and higher mortality (1,3). The complexity of RA arises from its multifactorial nature (4) and it has a heritability of approximately 60% (5).

OP is similarly multifactorial and demonstrates shared risk factors to RA (6). Dual X-ray absorptiometry is the standard technique used to measure bone mineral density (BMD) revealing OP and therefore fracture risk (7), though not the sole indicator of OP. In addition, bone architecture or quality is an important component of OP and fracture prediction (8). The heritability of BMD measures and fracture risk varies by skeletal site measured: 50–85 and 25–58%, respectively (9).

Consistent with previous studies including our own, the epidemiological association between RA and OP phenotypes is well established and has been observed across different samples considering various skeletal sites (10–12). The association between RA and OP is partially attributed to inflammatory factors altering bone resorption and leading to bone loss resulting from systemic inflammation seen in RA (13,14). C-reactive protein (CRP) is an inflammatory blood marker commonly used as a biomarker of RA; higher levels of CRP have been reported in subjects having both RA and OP in comparison to subjects with RA only (15). Yet, regardless of RA and independent of other covariates, an association between CRP and OP has been reported in a large sample (16). Moreover, a longitudinal study suggests that increased CRP levels predict faster BMD decline in women (17).

The three conditions—RA, OP and CRP—appear closely related. Thus, the extent to which genetic factors contribute to their correlation and the identification of shared genetic factors may shed light on pathogenic pathways of interest. Preliminary genetic analyses reported pleiotropy between RA and BMD (18) and between CRP and BMD (19). Still, our knowledge is virtually non-existent in regard to the genetic architecture behind the RA/OP/CRP trilateral relationship.

The main aim of this study was to explore the nature and possible causal genetic relationships between these two pathological musculoskeletal conditions and an inflammatory biomarker. Grounding on extensive clinical data, we explored the following hypotheses: RA, an inflammatory condition, necessarily contributes to raised CRP and this likely contributes to OP through bone turnover mechanisms and, therefore, the following sequence of pathological events is expected: RA → raised CRP; raised CRP→OP; RA→OP.

## Results

### Epidemiological assessment

The English Longitudinal Study of Ageing (ELSA) dataset comprised 4556 males and 5627 females of European ancestry with a mean age of 66 ± 10 and age range of 28–89 years. Binary multiple logistic regression analysis showed that RA was significantly and independently associated with OP [odds ratio (OR): 1.6627, 95% confidence interval (95% CI) = 1.1417–2.3604, *P* = 5.94E−03] and high-sensitivity CRP (hsCRP) (OR: 1.211, 95% CI = 1.0271–1.2082, *P* = 4.95E−03) regardless of age and body mass index (BMI) (Table 1). Sex, fibrinogen circulating levels and white blood cell count were also included at the entry stage as independent covariates of RA but were not retained in the final equation.

**Table 1:** Predictors of RA in ELSA

	RA-associated covariates						
Variables	B	SE	*z*-Value	*P*-value	OR	Lower 95% CI	Upper 95% CI
Intercept	−2.9581	0.0659	−44.9	<2.2E−16	0.0519	0.0455	0.0589
Age	0.2818	0.0650	4.3	1.47E−05	1.3254	1.1666	1.5056
BMI	0.3585	0.0534	6.7	2.01E−11	1.4311	1.2874	1.5877
hsCRP	0.1144	0.0407	2.8	4.95E−03	1.1211	1.0271	1.2082
OP	0.5084	0.1848	2.8	5.94E−03	1.6627	1.1417	2.3604

Multiple logistic regression on the ELSA dataset (Wave 6) where RA served as the dependent variable and OP served as an independent covariate in addition to age, BMI and hsCRP. The study sample is composed of 10 183 individuals in total of which 629 were diagnosed with RA and 786 were diagnosed as OP cases; hsCRP levels were measured in 6125 individuals. OR was estimated per SD of the phenotype variation. The independent variables initially included in the analysis were age, sex, BMI, hsCRP, fibrinogen levels, WBC count and OP. Only statistically significant results are reported in the table.

### Mendelian randomization analysis

To detect causality, RA served as the exposure variable, using single nucleotide polymorphisms (SNPs) from the meta-analysis as instruments, and a selected OP trait or CRP served as the outcome variable. Next, CRP served as the exposure variable and the selected OP trait as the outcome variable.

Results were considered valid if the heterogeneity assumption was not violated and *I^2^Gx* > 90% was achieved, thereby suggesting instrument variable (IV) strength and preserving Mendelian randomization (MR) assumptions. In addition to assessing causality and providing the causal estimate, the MR Egger (MRE) test estimates an intercept, which indicates horizontal pleiotropy if significant (*P* < 0.05).

#### RA→OP phenotypes

Implementing the inverse-variance-weighted (IVW) approach, RA appeared to causally predict OP and lower BMD measures (*P* < 0.001 for all; Supplementary Material, Table S1). Upon further investigation, using the MRE approach (Table 2), we clarified that RA appeared to be a causal risk factor for hip BMD (*β* = −0.036, 95% CI = −0.063 to −0.008, *P*-value = 1.10E−02), with no evidence of horizontal pleiotropy (*P*-value = 0.295). However, after Bonferroni correction for multiple comparisons, the association between RA and hip BMD did not achieve a significant MR estimate (*α* ≤ 0.00625) and, therefore, cannot be considered to be causal at this stage.

**Table 2:** MR of RA as exposure and OP or CRP as outcome using the MRE approach

Exposure→outcome	Number of IVs	MRE estimate	95% CI of MRE *β* value	MRE *P*-value	MR intercept *P*-value	Heterogeneity *P*-value	*I^2^Gx* (%)
RA→UKB OP	43	0.000	0.000, 0.001	0.603	0.001	0.9818	99.5
RA→OP fracture	34	0.014	−0.018, 0.037	0.495	<0.001	0.9483	99.2
RA→total body BMD	38	−0.005	−0.022, 0.012	0.562	0.002	0.8051	99.1
RA→spine BMD	43	0.006	−0.022, 0.034	0.680	0.001	0.9994	98.4
RA→hip BMD	41	−0.036	−0.063, −0.008	0.011	0.295	**0.7891**	96.9
RA→arm BMD	38	−0.021	−0.072, 0.030	0.418	0.006	0.9993	99.0
RA→UKB heel BMD	38	−0.006	−0.015, 0.003	0.200	<0.001	0.5505	99.1
RA→CRP	41	0.027	0.016, 0.038	<0.001	0.132	1.000	99.3

MRE results where RA served as the exposure variable with OP or CRP as the outcome variables in separate univariate analyses. The SNPs selected as instrumental variables had *P* ≤ 5E−08.

The other OP phenotypes did not show causal associations with RA, but each association revealed evidence of horizontal pleiotropy (Table 2). Specifically, in MR analysis of RA to OP fracture, the MRE intercept was highly statistically significant (*P*-value < 0.001), suggesting the presence of horizontal pleiotropy. Similarly, horizontal pleiotropy was apparent in the OP phenotype (*P* = 0.001) and each BMD assessment [total BMD *P* = 0.002, spine BMD *P* = 0.001, arm BMD *P* = 0.006 and UK Biobank (UKB) heel BMD *P* < 0.001].

#### RA→CRP

RA affection appeared causally and significantly predictive of increased levels of CRP, whether examining through the IVW approach (*β* = 0.033, 95% CI = 0.026–0.040, *P* < 0.001) (Supplementary Material, Table S1) or the MRE approach (*β* = 0.027, 95% CI = 0.016–0.038, *P* < 0.001) with no evidence of horizontal pleiotropy (*P* = 0.132) (Table 2).

#### CRP→OP phenotypes

In assessing IVW, elevated CRP appeared to be causally predictive of each OP trait tested (*P* ≤ 0.001 for all; Supplementary Material, Table S2). However, using the MRE approach, elevated CRP was not causally predictive of any OP phenotype tested; instead, horizontal pleiotropy appeared to be significantly involved in the association between elevated CRP and either the OP phenotype or OP fracture (*P* = 0.002 for both; Table 3). Similarly, BMD phenotypes displayed significant pleiotropic effects (total BMD *P* = 0.002, spine BMD *P* = 0.021, hip BMD *P* < 0.001, arm BMD *P* = 0.006 and UKB heel BMD *P* < 0.001; Table 3).

**Table 3:** MRE results where CRP was the exposure variable and OP served as the outcome variable in separate univariate analyses

Exposure→outcome	Number of IVs	MRE estimate	95% CI of MRE β value	MRE *P*-value	MR intercept *P*-value	Heterogeneity *P*-value	*I^2^Gx* (%)
CRP→UKB OP	32	−0.001	−0.003, 0.002	0.672	0.002	0.9676	98.7
CRP→OP fracture	25	−0.013	−0.116, 0.089	0.799	0.002	0.8983	98.1
CRP→total body BMD	44	−0.028	−0.080, 0.024	0.287	0.002	0.9465	98.3
CRP→spine BMD	40	−0.032	−0.110, 0.047	0.427	0.021	0.4210	98.8
CRP→hip BMD	41	0.006	−0.084, 0.095	0.896	<0.001	0.8518	98.0
CRP→arm BMD	37	−0.013	−0.187, 0.160	0.880	0.006	0.9964	98.0
CRP→UKB heel BMD	40	0.005	−0.021, 0.031	0.703	<0.001	0.9609	98.6

CRP served as the exposure variable and various OP phenotypes act as the outcome variables in separate univariate analyses. The SNPs selected as IVs had *P* ≤ 5E−08.

### Colocalization and gene-set enrichment analyses

While a causal relationship between CRP and OP, CRP– >OP phenotypes was not apparent, horizontal pleiotropy was consistently seen. In an attempt to identify potential common genes involved, we performed colocalization analyses, which provided significant strong (PP.H4 ≥ 75%) evidence of shared SNPs in five regions on chromosomes 1, 2, 9, 14 and 22 (Table 4). Moderate evidence of shared SNPs can be seen in Supplementary Material, Table S3, and low evidence of shared SNPs is observed in Supplementary Material, Table S4. The highest posterior probability (PP) of shared common SNPs (PP.H4) was observed between elevated CRP and total body BMD on chromosome 2 amounting to 99.3% and revealing an exonic non-synonymous SNP in gene *GCKR.* Comparable findings were detected between CRP and arm BMD and spine BMD, however, at lower PPs (PP.H4: 10.5 and 10.4%, respectively; Supplementary Material, Table S4). Subsequently, a 90.0% PP of shared common SNPs was observed between CRP and UKB heel BMD in chromosome 9 mapped to an ncRNA intronic gene, *ABO.* Similarly, strong evidence of shared SNPs was seen between CRP and several OP-related phenotypes: spine BMD, UKB OP and total body BMD on chromosome 14 (88.0, 84.7 and 84.4%, respectively). All were linked to the exonic non-synonymous SNPs in gene *SERPINA1* and the intergenic region of *SERPINA2P/SERPINA1* (Table 4). Colocalization was shown between UKB heel BMD and CRP only in an intergenic region of chromosome 1 around *LEPR/RN7SL854P* and an intergenic region in chromosome 2 around *AC093326.3/TMEM18* (PP.H4: 79.3 and 80.9%, respectively, Table 4). In addition, the genomic region of chromosome 22 demonstrated strong evidence of colocalizing SNPs between UKB heel BMD and CRP (76.4%; Table 4) and moderate evidence between OP fracture and CRP (73.0%; Supplementary Material, Table S3) corresponding to two genes, *GTPBP1* and *SUN2*. Similar observations between CRP and hip and spine BMD were seen on chromosome 22 but at much lower PPs (Supplementary Material, Table S4).

**Table 4:** Colocalization and gene-set enrichment for OP variables and CRP in H4

Chromosome and region	OP variable colocalized with CRP	Gene and (SNP)	Function	OP variable *P*-value	CRP *P*-value	PP. H4 (%)
Chr 1, 65 041 704–66 939 404	UKB heel QUS BMD	*LEPR/RN7SL854P* (rs13375019)	Intergenic	4.91E−05	1.35E−134	79.3
Chr 2, 10 133–1 781 022	UKB heel QUS BMD	*AC093326.3/TMEM18* (rs6548237)	Intergenic	4.86E−07	3.42E−08	80.9
Chr 2, 26 894 985–28 598 777	GEFOS total body BMD	*GCKR* (rs1260326)	Exonic, non-synonymous SNV, exon14	7.48E−08	5.44E−61	99.3
Chr 9, 135 298 842–137 041 122	UKB heel QUS BMD	*ABO* (rs507666)	ncRNA_intronic	1.71E−10	6.33E−09	90.0
Chr 14, 94 325 285–95 750 867	UKB OP	*SERPINA1* (rs28929474)	Exonic, non-synonymous SNV, exon5	2.25E−05	5.47E−10	84.7
Chr 14, 94 325 285–95 750 867	UKB OP	*SERPINA2P/SERPINA1* (rs112635299)	Intergenic	1.43E−05	2.20E−10	84.7
Chr 14, 94 325 285–95 750 867	GEFOS total body BMD	*SERPINA1* (rs28929474)	Exonic, non-synonymous SNV, exon5	3.65E−04	5.47E−10	84.4
Chr 14, 94 325 285–95 750 867	GEFOS total body BMD	*SERPINA2P/SERPINA1* (rs112635299)	Intergenic	4.27E−04	2.20E−10	84.4
Chr 14, 94 325 285–95 750 867	GEFOS spine BMD	*SERPINA2P/SERPINA1* (rs112635299)	Intergenic	3.66E−04	2.20E−10	88.0
Chr 22, 37 570 269–39 307 894	UKB heel QUS BMD	*SUN2* (rs1062687)	Exonic, synonymous SNV, exon16	4.31E−09	2.97E−09	76.4
Chr 22, 37 570 269–39 307 894	UKB heel QUS BMD	*GTPBP1* (rs2267395)	Intronic	6.59E−09	4.75E−10	76.4

Results of common colocalizing genetic variants and their respective genes seen between OP phenotypes and CRP as determined by high PP of H4 (PP.H4 ≥ 75%) arising from commonly shared SNPs.

Other colocalization regions with a moderate PP.H4 (between 50 and 75%) were found on chromosomes 8 and 1: between spine BMD and CRP in the intronic regions of *RP11-115J16.1* and of *RP11-115J16.1* (PP.H4: 61.0%; Supplementary Material, Table S3) and the ncRNA intronic region belonging to *PPIEL* (chromosome 1) (PP.H4: 59.0%) between OP fracture and elevated CRP (SupplementaryMaterial, Table S3). Colocalization results showing evidence of PP < 50% are given in Supplementary Material, Table S4.

To confirm the pleiotropic relationship between CRP and OP traits seen in Table 4, attributed to SNPs with high PP.H4, the PLeiotropic Analysis under COmposite null hypothesis (PLACO) analysis was carried out. The PLACO analysis revealed that each tested SNP was highly significantly pleiotropic between CRP and the designated OP variable, and the corresponding *P*-values ranged between 1.06E−09 and 1.17E−35 (Supplementary Material, Table S5).

In assessing the PP of H3 (PP.H3) between CRP and OP fracture and several BMD phenotypes in different study samples, 23 regions were revealed with high PP of at least 80%, though most achieving nearly 100% of colocalization arising from distinct SNPs. All of the results are provided in Supplementary Material, Table S6.

Gene enrichment results, denoted as genes of interest, were only reported for findings consistent with those in Table 4 and were attributed to high PP.H4, or genes with small *P*-values and PP.H3s approaching 100% (Supplementary Material, Table S7), since high PP.H3s may also be owing to spurious pleiotropy.

Notable for its reoccurrence, high PP.H3s between both UKB heel BMD and total BMD with CRP (PP.H3: 95.6% and 99.6%, respectively) revealed an exonic non-synonymous SNP in the *SERPINA1* gene around the intergenic region of *SERPINA2P/SERPINA1* on chromosome 14 (Supplementary Material, Table S7).

We further examined potential colocalizing regions between RA and CRP. Two regions of interest on chromosome 6 were apparent. The first region is between base pairs 30 798 168 and 31 571 218, with a PP of distinct SNPs of 93.1% and linked to the intergenic region of *HCP5/MICB* (Supplementary Material, Table S8). The second region with PP.H3 of 69.2% was found between the base pairs 31 571 218 and 32 682 664 in the intergenic region of *HLA-DRB1/HLA-DQA1*.

### Gene ontology

Since the colocalization analysis revealed a number of exonic SNPs that were significant in CRP and OP phenotypes, we examined whether they could be assigned to a set of genetic factors connected by a common pathway or share a gene ontology (GO) term denoting a common genetic topology. GO enrichment analysis was carried out for RA, CRP and all OP phenotypes. Next, the GO terms were mapped and annotated per phenotype and were further assessed using Fisher’s exact test to generate top scores for GO terms present per selected class. Conversely, we identified whether at least one GO term included *SERPINA1* and/or *GCKR* and observed that only *GO:0005515* term describes both genes.

Fisher’s test conducted for the GO molecular functions class using the classic algorithm revealed that *GO:0005515* is significantly over-represented in SNPs associated with RA (*P* = 3.30E−10), CRP (*P* = 5.90E−07) and several OP traits, including UKB OP (*P* = 1.40E−15), total BMD (*P* = 2.40E−14), spine BMD (*P* = 1.40E−19), hip BMD (*P* = 1.20E−10) and UKB heel BMD (*P* = 3.10E−06), annotated over 13 000 genes (Supplementary Material, Table S9). Arm BMD and OP fracture variables of the GEnetic Factors for OSteoporosis (GEFOS) Consortium did not reveal significant associations with the GO term examined. Supplementary Material, Table S9 summarizes the ranking of the node, the respective annotations and Fisher’s tests. *GO:0005515* term was among the top three nodes for each variable that was significantly associated under the molecular functional class. The other two classes did not display statistically significant findings. *GO:0005515* denotes protein binding and, therefore, demonstrates that RA, OP and CRP share a common genetic pathway that is involved in the molecular functional level of protein binding. The GO universe that explained this particular GO term is associated with both the *SERPINA1* and *GCKR* genes (along with another ~13 000 genes), which showed high colocalization between CRP and OP phenotypes and revealed exonic SNPs in both genes.

In addition to the two major exonic SNPs that were highly colocalized between CRP and OP phenotypes, we identified genes demonstrating colocalization between CRP and OP arising from common and distinct SNPs (Table 4 and Supplementary Material, Table S7) and were also a component of *GO:0005515*. We observed several genes that contribute to *GO:0005515* from chromosomes 1, 2, 8, 11, 16, 18 and 22, including *LEPR1*, *TMEM18*, *PPP1CB*, *SPDYA*, *TRMT61B*, *MSRA*, *TRPS1*, *C11orf49*, *FTO*, *PTPN2*, *LDLRAD4*, *RNMT*, *GTPBP1* and *SUN2.* We also examined genes in colocalization between RA and CRP and found two additional genes that are components of *GO:0005515*, including *HLA-DRB1* and *HLA-DQA1*.

## Discussion

The main aim of this study was to clarify the relationship between RA and OP and to additionally clarify whether increased CRP serves a role in the relationship and to what extent it is grounded on clinical data. The latter suggests that RA, an inflammatory condition, necessarily contributes to raised CRP and this likely leads to OP through bone turnover mechanisms (17). These aims were accomplished by implementing MR, which ascertains whether a causal relationship is present and may explain the relationship between RA and OP. In addition, we determined which genes are involved in the pleiotropic relationship that appeared between CRP and OP.

MRE analyses between RA and varying OP phenotypes were suggestive of pleiotropy, which was previously shown by us (20) using polygenic risk score analysis in addition to colocalization and gene enrichment analyses and it is consistent with previous reports (18,21). Several loci have been discovered in relation to both phenotypes, particularly in the major histocompatibility complex (18,21). However, these pleiotropic regions appear to be polygenic with no clear contributing SNP. Here, we confirmed that the suggested common causal SNPs were pleiotropic through assessing PLACO.

Risk factors common to both RA and OP are well known (22) and include heightened RA disease activity, increased RA disease duration and the quality of life of RA subjects (12,23) and showed that RA subjects were at a greater risk for overall bone fracture and fragility fracture at the vertebral, hip, forearm and proximal humerus (23). However, recent studies highlighted the inflammatory component involved in the relationship between RA and OP, which is exacerbated with RA disease activity (14,22,24).

Elevated CRP is a response to systemic inflammation elicited by RA and is correlated with other RA disease markers, including bone and joint erosion, early morning stiffness and swollen joints and, in particular, bone deterioration (25). As such, MR results where RA causally predicts CRP are expected.

Importantly, however, our data consistently indicated that CRP–OP relationship is mostly attributable to horizontal pleiotropy. A shared genetic profile between CRP and OP traits was suggestive using PLACO and was subsequently confirmed using MRE, further suggesting that pleiotropy may explain the genetic relationship between CRP and OP.

The suspected pathophysiology, including CRP elevation in RA subjects, was suggested to arise from prompting *RANKL* as well as osteoclastogenesis, thereby eliciting bone resorption and subsequent BMD deterioration (25,26). The present results further clarify the strong and complex genetic relationship between RA and OP, which likely occurs via elevated CRP.

We implemented a colocalization analysis and functional annotation to investigate potential pleiotropic genes between CRP and OP focusing on non-synonymous SNPs, which are potentially deleterious for protein structure and function, in addition to demonstrating a high degree of colocalization. Two major non-synonymous SNPs of genes, *GCKR* and *SERPINA1,* emerged consistently between CRP and various OP phenotypes. A previous study similarly demonstrated that hsCRP and OP phenotypes do not share a causal association; however, the study also identified *GCKR* and *ABO* as pleiotropic genes (19). Though this appears consistent with our findings, *ABO* was considered to be associated with interleukin-6, a different but closely linked inflammatory marker (19). *GCKR* codes for a glucokinase regulator, which is involved in metabolic modification and is associated with several phenotypes, including bone and inflammatory phenotypes (27). *SERPINA1* is influenced by *GCKR* and is similarly involved in metabolic alteration and suggestively contributes to the pathway involving metabolic aberration through inflammatory mechanisms arising from alpha-1 anti-trypsin deficiency (28). Taken together, these genes, which were prominent and pleiotropic across CRP and OP, suggest a potential pathway involving metabolic syndrome. Perhaps, these genes may be influenced by BMI and other latent variable(s), which may mediate the relationship between CRP and OP phenotypes. PPs were not strong in examining all OP phenotypes; however, differences among OP phenotypes are most probably attributable to smaller sample sizes, resulting in an under-powered assessment.

The HLA region was suggestive of pleiotropy between RA and CRP, specifically genes *HCP5/MICB* and region *HLA-DRB1/HLA-DQA1.* RA with high inflammatory markers in the presence of *HLA-DRB1* alleles was shown to have a greater risk of cardiovascular complications (29) and increased disease severity of RA (30).

To summarize the complex genetic architecture connecting RA and OP, we propose a simplified model that explains these relations. We speculate that while a portion of the genetic influence in RA and OP manifestation may be phenotype-specific, pleiotropic genes contribute polygenically to the development of RA and OP. In addition, elevated CRP results from systemic inflammation, in this case from RA, and is pleiotropically associated with low BMD and OP fracture. The role of CRP further demonstrates the complex multifactorial nature of RA and RA-induced OP.

### Limitations

We limited our genome-wide association study (GWAS) datasets to samples of European ancestry, thus our findings may not be applicable to the global population. The GEFOS OP fracture dataset includes data from the UKB, which may lead to bias and overinflated results. While the use of GWAS datasets from differing data sources may contribute to reducing power, it allows for limiting bias in a two-sample MR study. The effect of behavior and lifestyle among other potential confounders cannot be evaluated through MR studies. We could not determine SNP independence prior to conducting MR analyses since the RA phenotype is binary in nature, so we were unable to extract SNPs that may serve as confounders. Despite this limitation, MR findings were similar across different BMD GWAS summary statistics. Different BMD samples were used, as well as two different BMD measurements with different sample sizes, which lends strength to the findings, while recognizing that the anatomical sites show differing susceptibility to OP. The SNPs identified in GWAS are not necessarily the true causal SNPs. Thus, fine mapping would be advantageous to determine the extent to which the SNPs alter or contribute to the phenotype.

### Concluding remarks

The complex relationship between RA and OP inclusive of inflammatory mechanisms should be clarified using molecular techniques. These three phenotypes share genetic factors that play a role in protein binding. Further investigation may reveal the intricacies of the shared molecular pathway potentially involved in the development of the debilitating musculoskeletal conditions and may lead to insight regarding pathways and potential therapeutics.

## Materials and Methods

### Study design

We conducted analyses using several large datasets and implemented modern genetic epidemiology techniques involving several consecutive analytical steps:

(1) We established the relationship between the three variables of interest (RA, OP and CRP) independent of possible confounders.
(2) We estimated causality between the study phenotypes, assuming that RA is the causal factor for OP and elevated CRP; CRP is causal factor for OP.
(3) We examined the shared genomic regions for colocalization to reveal pleiotropy while conducting gene-set enrichment analysis to identify the potential common genes.
(4) We confirmed pleiotropic effects for SNPs shared by CRP and OP traits, which showed high PP of being causal to both traits using a statistical pleiotropy test, PLACO.
(5) We performed GO analysis to describe common genetic pathways.

### Dataset for phenomenological analysis

A cross-sectional study was performed using data from the ELSA Wave 6 (31). The dataset consisted of 10 183 participants of European descent (4744 males and 5857 females), including 7731 individuals with measurements of hsCRP among other serological markers, collected during 2012 and 2013. Of these, 661 participants were diagnosed with RA and 819 with OP.

### Datasets for genetic analysis

In total, 11 GWAS datasets were used. Summary statistics for GWAS conducted by the Neale Laboratory (32) using UKB phenotypes, including RA [5422 cases and 355 719 controls (binary)], OP [7734 cases and 353 407 controls (binary)], calcaneus BMD measured by quantitative ultrasound (QUS) [*n* = 206 496 (quantitative)] and CRP [*n* = 343 524 (quantitative)], were downloaded from the Pan UKBB website (https://pan.ukbb.broadinstitute.org). Each GWAS embraced over 13 million genotyped and imputed genetic variants, SNPs and indels.

We collected additional GWAS summary statistics to conduct genetic analyses in a two-sample setting whenever applicable. We obtained another RA GWAS conducted by Okada et al. (33), which consisted of meta-analyzed data and was restricted to subjects of European ancestry (33) and comprised of 14 361 RA cases and 43 923 controls from 18 data sources with over 8 million imputed SNPs. Each RA subject was diagnosed in accordance with the revised 1987 criteria of the American Rheumatism Association or by a professional rheumatologist.

Meta-analyzed BMD GWAS (quantitative variables) and OP fracture summary statistics (binary variable) were acquired from the GEFOS consortium (http://www.gefos.org/) and contained BMD measures of the forearm (*n* = 8143), hip femoral neck (*n* = 32 735), lumbar spine (*n* = 28 498) (34) and life course total body (*n* = 66 628) (35). In addition, a large-scale OP fracture GWAS (37 857 cases and 264 973 controls) was also acquired (36). The GWAS summary statistics for arm, hip and spine BMD consisted of nearly 10 million imputed SNPs. The GWAS summary statistics of total body BMD contained over 18 million SNPs, and for OP fracture, over 2.5 million SNPs.

Finally, CRP GWAS summary statistics (quantitative variable) were obtained from the Cohorts for Heart and Aging Research (CHARGE) consortium (https://www.chargeconsortium.com/) Inflammation Working Group (37) and it encompassed 204 402 participants gathered from 78 studies and 10 million SNPs.

### Statistical analysis

#### Association between RA, OP and CRP

Statistical assessment of the ELSA phenotypes was conducted using R software environment (http://www.R-project.org/) to determine the extent of the association of RA (as the dependent variable) with OP and CRP circulating levels (the independent variables) in addition to covariates age, sex and BMI. The glm function from the R stats package was used to conduct logistic regression analysis, implementing the pairwise deletion approach in case of missing data.

#### Mendelian randomization

**Figure 1:**
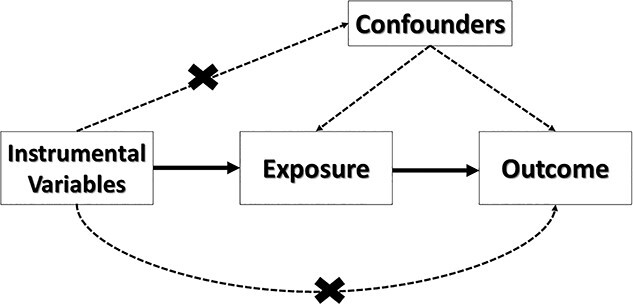
The diagram represents the three MR assumptions: (1) the IVs or genetic variants (SNPs of interest) are associated with the exposure phenotype only, (2) the exposure and outcome do not share a common cause (confounders) and (3) the IVs influence the outcome only through the exposure.

To test for the potential causal effect between two phenotypes, we performed MR analysis using GWAS summary statistics and employed the MendelianRandomization package in R (38). Figure 1 describes the MR methodology.

Several MR approaches are available with the intent to satisfy different assumptions, including IVW and MRE (39). Except for MRE, we found most other MR approaches to correspond closely with IVW. Thus, we initially focused on MRE and IVW approaches.

The MRE approach is advantageous because it is a robust approach that assumes and detects pleiotropy (40). While it is more conservative, it provides a more pronounced bias in a two-sample setting with overlap or one-sample setting, as it is prone to type I error. A component of the MRE approach determines instrument validity, the *I^2^Gx* sensitivity statistic, which was used to measure the instrumental variable dilution or bias (41), and it was suggested to be at least 90% in a two-sample analysis (40).

IVs, or genetic variants of the causal trait, were chosen following the compilation of the GWAS summary statistics of the exposure and outcome phenotypes. The *P*-value threshold of the effect of the exposure variable was restricted to *P* < 5E−08 regardless of the *P*-value attributed to the outcome phenotype. Subsequently, linkage disequilibrium (LD) clumping was performed to remove SNPs in LD by utilizing the ld_clump function available in the MRCIEU/ieugwasr R package (42) with a parameter of clumping *R*^2^ = 0.01. This procedure along with the harmonization of the GWAS summary statistics of the two traits being compared were performed (43). In assessing RA/CRP as a risk factor for BMD, IVs were restricted to associated beta estimates of opposing signs between the two traits; conversely, in assessing whether RA is a risk factor of OP binary phenotypes or CRP or if CRP is a risk factor of OP binary phenotypes, IVs were restricted to associated beta estimates of the same signs for both traits.

We chose the meta-analyzed RA GWAS and CHARGE CRP GWAS instead of their UKB GWAS counterparts since we preferred to perform two-sample MR tests while avoiding sample overlap.

#### Colocalization analysis and gene enrichment analysis

Pleiotropy may explain the genetic relationship between OP phenotypes and CRP, so we conducted colocalization analysis to further decipher the regions that may contain shared SNPs (44). We examined SNPs that appeared to colocalize either through mutual common SNPs or distinct SNPs within the same gene. The colco.abf function from the coloc package in R was used (45). This colocalization method is based on Bayesian modeling and includes testing of the PPs of five mutually exclusive hypotheses (44). We scanned for SNPs achieving GWAS significance threshold for CRP and defined the corresponding genomic region for colocalization analysis by LD Detect blocks (46). LD detect regions were selected as boundaries of prominent SNPs, which include generated independent LD blocs (46). These boundaries are the regions where a higher frequency of recombination takes place. Subsequently, the PPs of each of the five hypotheses were evaluated in relation to OP phenotypes.

We focused on Hypothesis 4 (H4) and Hypothesis 3 (H3), which assume the existence of causal SNP(s) attributed to both traits in a given genomic region. However, H4 estimates the PP of shared common SNPs affecting both phenotypes, whereas H3 estimates the PP of distinct SNPs within the same region, although common SNPs may also be present (44). The PP was considered as having strong evidence if it was at least 75%, while a PP of at least 50% was cautiously suggestive of evidence of different causal SNPs mapped to the designated genomic region (45).

The coloc.abf function additionally generates an output with SNP.PP.H4, which includes the PP of each SNP being the causally conditional shared SNP, corresponding to H4, between the two examined traits. Following a PP of H4 > 50%, we focused on the SNP with the highest PP to explain the suggested colocalized relationship, while prioritizing and/or also reporting exonic SNPs.

Next, we used the functional mapping and annotation GWAS platform (47) for gene enrichment analysis to define the genes in common between the two phenotypes, while focusing on SNPs with the smallest *P*-value for both traits.

#### Statistical presence of pleiotropy

PLACO was implemented to determine evidence of pleiotropy of the selected shared SNP between two traits (48). SNPs were identified through the colocalization analysis and were subsequently assessed using PLACO to confirm the pleiotropic relationship between the traits, which was suggestively attributed to the SNP. A significant *P*-value from PLACO is suggestive of a common genetic framework between the traits. PLACO utilizes GWAS summary statistics to generate the pleiotropic test, which is subsequently tested considering the *Z* scores (beta/effect) of each trait in relation to the SNP in question (48). PLACO is limited to common causal SNPs and was, therefore, only applied to SNPs showing high PP of H4 from the colocalization analysis.

#### Gene ontology

GO enrichment analysis was carried out to decipher at least one potential model that would describe genetic topology of one GO term or node and can be calculated under three classes: biological process, molecular function and cellular component (49). Each GO term explains a cluster of genes that contribute to the process, function or component examined. Thus, we used the topGO R package in Bioconductor (50) to identify highly associated GO terms under the three classes with RA, OP and CRP variables. Algorithmic parameters were set to ‘classic’, and Fisher’s exact test statistic was assessed.

## Data availability

Data were acquired from databases and consortia ,such as GEFOS, UKB, Neale Laboratory, CHARGE and ELSA. Access to these data may be provided from the aforementioned sources.

## Supplementary Material

3_RA_OP_MR_Supplementary_Material_Resubmission_Final_ddac061
