# Supplementary material for: Understanding the complex genetic architecture connecting rheumatoid arthritis, osteoporosis and inflammation: discovering causal pathways
Source: Hum Mol Genet. 2022 Mar 29;31(16):2810–9. doi: 10.1093/hmg/ddac061 (PMC9402243; doi:10.1093/hmg/ddac061)
Supplement: 3_RA_OP_MR_Supplementary_Material_Resubmission_Final_ddac061 [file 3_ra_op_mr_supplementary_material_resubmission_final_ddac061.docx]

**Understanding the complex genetic architecture connecting rheumatoid arthritis, osteoporosis, and inflammation: Discovering causal pathways**

**Supplementary Material**

Melody Kasher^1^, Frances M.K. Williams^2^, Maxim B. Freidin^2^, Ida Malkin^1^, Stacey S. Cherny^1, 3^, CHARGE Inflammation Working Group, Gregory Livshits^1, 2, 4 *^

1. Human Population Biology Research Unit, Department of Anatomy and Anthropology, Sackler Faculty of Medicine, Tel Aviv University, Israel

2. Department of Twin Research and Genetic Epidemiology, School of Life Course Sciences, King’s College London, London, UK

3. Department of Epidemiology and Preventive Medicine, Sackler Faculty of Medicine, Tel Aviv University, Tel Aviv, Israel

4. Adelson Medical School, Ariel University, Ariel, Israel

* Corresponding author:

Department of Anatomy and Anthropology,

Sackler Faculty of Medicine, Tel-Aviv University,

Ramat-Aviv, Tel-Aviv, Israel.

Telephone: 972-3640-9494

Email: [gregl@tauex.tau.ac.il](mailto:gregl@tauex.tau.ac.il)

**Table ST1:** Mendelian Randomization of RA as exposure and OP or CRP as outcome using the IVW approach

| Variables | No of IVs | IVW Estimate | 95% CI of  β value | p-value | Hetero-geneity  p-value |
| --- | --- | --- | --- | --- | --- |
| RA🡪UKB OP | 43 | 0.001 | 0.001, 0.001 | <0.001 | 0.7585 |
| RA🡪 OP fracture | 34 | 0.048 | 0.030, 0.065 | <0.001 | 0.4951 |
| RA🡪 total body BMD | 38 | -0.025 | -0.036, -0.014 | <0.001 | 0.4140 |
| RA🡪 spine BMD | 43 | -0.032 | -0.048, -0.015 | <0.001 | 0.9423 |
| RA🡪 hip BMD | 41 | -0.048 | -0.063, -0.033 | <0.001 | 0.7821 |
| RA🡪 arm BMD | 38 | -0.076 | -0.109, -0.043 | <0.001 | 0.9701 |
| RA🡪UKB heel BMD | 38 | -0.020 | -0.026, -0.014 | <0.001 | 0.1262 |
| RA🡪 CRP | 41 | 0.033 | 0.026, 0.040 | <0.001 | 0.9722 |

Legend to Table ST1

IVW MR results where RA served as the exposure variable with OP or CRP as the outcome variables, in separate univariate analyses. The SNPs selected as IVs had p ≤5E-08.

**Table ST2:** Mendelian Randomization of CRP as exposure and OP as outcome using the IVW approach

| Variables | No of IVs | IVW Estimate | 95% CI of MR  β value | p-value | Hetero-geneity  p-value |
| --- | --- | --- | --- | --- | --- |
| CRP🡪UKB OP | 32 | 0.003 | 0.001, 0.005 | 0.001 | 0.6893 |
| CRP🡪 OP fracture | 25 | 0.110 | 0.033, 0.045 | 0.001 | 0.4585 |
| CRP🡪 total body BMD | 44 | -0.091 | -0.124, -0.057 | <0.001 | 0.7050 |
| CRP🡪 spine BMD | 40 | -0.103 | -0.156, -0.051 | <0.001 | 0.2495 |
| CRP🡪 hip BMD | 41 | -0.135 | -0.188, -0.083 | <0.001 | 0.3096 |
| CRP🡪 arm BMD | 37 | -0.215 | -0.318, -0.113 | <0.001 | 0.9241 |
| CRP🡪UKB heel BMD | 40 | -0.042 | -0.061, -0.024 | <0.001 | 0.2021 |

Legend to Table ST2

IVW MR results where CRP served as the exposure variable with OP traits as the outcome variables, in separate univariate analyses. The SNPs selected as IVs had p ≤5E-08.

**Table ST3**: Causal colocalizing genes between OP traits and CRP as determined by posterior probability of H4 (75%>PP.H4>50%)

| Chromosome & region | OP variable colocalized with CRP | Gene and  (SNP) | Function | OP variable p-value | CRP p-value | PP. H4 |
| --- | --- | --- | --- | --- | --- | --- |
| Chr 1, 38731847-40200567 | **GEFOS OP fracture** | *PPIEL* (rs4660808) | ncRNA_intronic | 5.46E-04 | 7.48E-14 | 59.0% |
| Chr 8, 7153079-9154694 | **GEFOS spine BMD** | *RP11-115J16.1* (rs330088) | ncRNA_intronic | 6.23E-04 | 4.75E-15 | 63.9% |
| Chr 8, 7153079-9154694 | **GEFOS hip BMD** | *RP11-115J16.1* (rs330088) | ncRNA_intronic | 5.74E-04 | 4.75E-15 | 60.1% |
| Chr 22, 37570269-39307894 | **GEFOS OP fracture** | *SUN2* (rs6001214) | intronic | 1.88E-04 | 9.89E-10 | 73.0% |
| Chr 22, 37570269-39307894 | **GEFOS OP fracture** | *GTPBP1* (rs2267395) | intronic | 4.95E-04 | 4.75E-10 | 73.0% |

Legend to Table ST3: Results of common colocalizing genetic variants and their respective genes seen between OP phenotypes and CRP as determined by high posterior probability of H4 (between 50% and 75%) arising from common shared SNPs

**Table ST4**: Causal colocalizing genes between OP traits and CRP as determined by posterior probability of H4 (PP.H4<50%)

| Chromosome and Region | OP phenotype colocalized with CRP | Gene (Symbol) and SNP | Function | OP Phenotype p-value | CRP p-value | Posterior Probability H4 |
| --- | --- | --- | --- | --- | --- | --- |
| Chr 1, 38731847-40200567 | GEFOS spine BMD | *MACF1* (rs113603865) | intronic | 8.18E-04 | 1.53E-11 | 27.1% |
| Chr 1, 38731847-40200567 | GEFOS spine BMD | *PPIEL* (rs4660808) | ncRNA_intronic | 2.88E-03 | 7.48E-14 | 27.1% |
| Chr 1, 90066303-91886317 | GEFOS arm BMD | *RPL5P6*  *HFM1* (rs469773) | intergenic | 8.15E-03 | 2.99E-11 | 17.1% |
| Chr 2, 26894985-28598777 | GEFOS spine BMD | *GCKR* (rs1260326) | exonic, nonsynonymous SNV, exon14 | 1.29E-02 | 5.44E-61 | 10.4% |
| Chr 2, 26894985-28598777 | GEFOS arm BMD | *GCKR* (rs1260326) | exonic, nonsynonymous SNV, exon14 | 2.28E-02 | 5.44E-61 | 10.5% |
| Chr 3, 49316972-51832015 | UKB heel QUS BMD | *IP6K1* (rs3749237) | intronic | 1.34E-13 | 5.81E-09 | 44.9% |
| Chr 3, 49316972-51832015 | UKB heel QUS BMD | *CDHR4* (rs71324996) | intronic | 7.23E-14 | 1.31E-09 | 44.9% |
| Chr 3, 49316972-51832015 | UKB heel QUS BMD | *UBA7* (rs1799845) | intronic | 2.14E-13 | 1.72E-09 | 44.9% |
| Chr 3, 49316972-51832015 | UKB heel QUS BMD | *TRAIP* (rs10049413) | intronic | 1.98E-13 | 3.69E-10 | 44.9% |
| Chr 5, 171074292-172678327 | GEFOS hip BMD | *DUSP1* (rs34471628) | exonic, nonsynonymous SNV, exon3 | 2.72E-02 | 3.71E-11 | 10.5% |
| Chr 6, 31571218- 32682664 | GEFOS OP fracture | *BTNL2* (rs3793127) | intronic | 4.74E-03 | 2.93E-08 | 12.6% |
| Chr 7, 22507629-23471442 | GEFOS arm BMD | *AC002480.2/*  *AC073072.5* (rs62447656) | intergenic | 1.75E-03 | 9.14E-13 | 47.3% |
| Chr 8, 7153079-9154694 | GEFOS OP fracture | *RP11-115J16.1* (rs17658270) | ncRNA_intronic | 5.14E-04 | 1.17E-12 | 16.7% |
| Chr 8, 10463197-11278998 | GEFOS OP fracture | *LINC00529* (rs10481454) | ncRNA_intronic | 6.11E-04 | 1.29E-10 | 21.6% |
| Chr 8, 10463197-11278998 | UKB heel QUS BMD | *LINC00529* (rs10481454) | ncRNA_intronic | 3.60E-36 | 1.29E-10 | 33.5% |
| Chr 8, 10463197-11278998 | UKB heel QUS BMD | *LINC00208* (rs2898290) | ncRNA_exonic | 1.40E-36 | 1.29E-10 | 33.5% |
| Chr 8, 11278998-13491775 | GEFOS OP fracture | *Not shown in FUMA assessment* | N/A | N/A | N/A | 22.0% |
| Chr 8, 126410917-128659111 | GEFOS arm BMD | *RP11-136O12.2* (rs10956251) | ncRNA_intronic | 1.82E-02 | 2.39E-09 | 12.0% |
| Chr 14, 94325285-95750867 | GEFOS hip BMD | *SERPINA2P/*  *SERPINA1* (rs112635299) | intergenic | 1.79E-02 | 2.20E-10 | 10.3% |
| Chr 16, 49007926-52035823 | GEFOS arm BMD | *SALL1* (rs116971887) | UTR3 | 2.22E-02 | 2.33E-20 | 13.0% |
| Chr 20, 42680176-44839056 | GEFOS spine BMD | *HNF4A* (rs1800961) | exonic, nonsynonymous SNV, exon4 | 7.54E-03 | 2.38E-15 | 30.4% |
| Chr 22, 37570269-39307894 | GEFOS spine BMD | *GTPBP1* (rs2267395) | intronic | 1.81E-02 | 4.75E-10 | 15.0% |
| Chr 22, 37570269-39307894 | GEFOS spine BMD | *SUN2* (rs1062687) | exonic, synonymous SNV, exon16 | 2.77E-02 | 2.97E-09 | 15.0% |
| Chr 22, 37570269-39307894 | GEFOS hip BMD | *GTPBP1* (rs2267393) | intronic | 3.93E-02 | 8.61E-10 | 12.9% |
| Chr 22, 37570269-39307894 | GEFOS hip BMD | *SUN2* (rs2072794) | intronic | 4.14E-03 | 1.32E-09 | 12.9% |

Legend to table ST4: Results of common colocalizing genetic variants and their respective genes seen between OP phenotypes and CRP as determined by a lower posterior probability of H4 arising from shared causal SNPs

**Table ST5**: PLACO results depicting significant pleiotropy between CRP and OP trait in question that demonstrated moderate to high PP of H4, shared causal SNPs.

| Chromosome & region | OP variable colocalized with CRP | Gene and  (SNP) | T.PLACO | P.PLACO | PP. H4 |
| --- | --- | --- | --- | --- | --- |
| Chr 1, 38731847-40200567 | **GEFOS OP fracture** | *PPIEL* (rs4660808) | -25.95 | 4.75E-11 | 59.0% |
| Chr 1, 65041704-66939404 | **UKB heel QUS BMD** | *LEPR/RN7SL854P* (rs13375019) | -100.24 | 5.43E-33 | 79.3% |
| Chr 2,  10133-1781022 | **UKB heel QUS BMD** | *AC093326.3/TMEM18* (rs6548237) | 27.77 | 3.89E-10 | 80.9% |
| Chr 2, 26894985-28598777 | **GEFOS total body BMD** | *GCKR* (rs1260326) | -89.14 | 1.17E-35 | 99.3% |
| Chr 8, 7153079-9154694 | **GEFOS spine BMD** | *RP11-115J16.1* (rs330088) | 27.45 | 7.25E-12 | 63.9% |
| Chr 8, 7153079-9154694 | **GEFOS hip BMD** | *RP11-115J16.1* (rs330088) | 27.69 | 5.86E-12 | 60.1% |
| Chr 9, 135298842-137041122 | **UKB heel QUS BMD** | *ABO* (rs507666) | -37.08 | 4.21E-13 | 90.0% |
| Chr 14, 94325285-95750867 | **UKB OP** | *SERPINA1* (rs28929474) | -26.30 | 1.77E-11 | 84.7% |
| Chr 14, 94325285-95750867 | **UKB OP** | *SERPINA2P/SERPINA1* (rs112635299) | -27.54 | 5.86E-12 | 84.7% |
| Chr 14, 94325285-95750867 | **GEFOS total body BMD** | *SERPINA1* (rs28929474) | 22.09 | 1.06E-09 | 84.4% |
| Chr 14, 94325285-95750867 | **GEFOS total body BMD** | *SERPINA2P/SERPINA1* (rs112635299) | 22.34 | 8.48E-10 | 84.4% |
| Chr 14, 94325285-95750867 | **GEFOS spine BMD** | *SERPINA2P/SERPINA1* (rs112635299) | 23.16 | 3.38E-10 | 88.0% |
| Chr 22, 37570269-39307894 | **GEFOS OP fracture** | *SUN2* (rs6001214) | -22.86 | 7.06E-10 | 73.0% |
| Chr 22, 37570269-39307894 | **GEFOS OP fracture** | *GTPBP1* (rs2267395) | -21.73 | 1.88E-09 | 73.0% |
| Chr 22, 37570269-39307894 | **UKB heel QUS BMD** | *SUN2* (rs1062687) | 34.84 | 2.18E-12 | 76.4% |
| Chr 22, 37570269-39307894 | **UKB heel QUS BMD** | *GTPBP1* (rs2267395) | 36.13 | 8.49E-13 | 76.4% |

Legend to table ST5: T.PLACO represents the t-test results of the PLACO analysis and P.PLACO represents the corresponding p-value.

**Table ST6:** Genomic Regions with a high probability of postulate H3, where all other posterior probabilities fall below <0.10, defining the probability of shared distinct causal SNPs apparent in the given genomic region.

| Genomic Region (CHR & BP) | Osteoporotic phenotype in colocalization with CRP | PP.H3.abf |
| --- | --- | --- |
| Chr1 25516845-27401867 | UK Biobank heel QUS BMD | 9.30E-01 |
| Chr2 28598777-29217559 | UK Biobank heel QUS BMD | 9.71E-01 |
| Chr2 110572432-113921856 | UK Biobank heel QUS BMD | 9.84E-01 |
| Chr6 31571218-32682664 | UK Biobank heel QUS BMD | 9.27E-01 |
| Chr6 31571218-32682664 | GEFOS hip BMD | 8.98E-01 |
| Chr6 125424383-127540461 | GEFOS OP fracture | 1.00E+00 |
| Chr6 125424383-127540461 | UK Biobank heel QUS BMD | 1.00E+00 |
| Chr6 125424383-127540461 | GEFOS total body BMD | 9.88E-01 |
| Chr6 125424383-127540461 | GEFOS hip BMD | 9.61E-01 |
| Chr8 7153079-9154694 | UK Biobank heel QUS BMD | 9.70E-01 |
| Chr8 9154694-9640787 | UK Biobank heel QUS BMD | 1.00E+00 |
| Chr8 9640787-10463197 | UK Biobank heel QUS BMD | 9.34E-01 |
| Chr8 11278998-13491775 | UK Biobank heel QUS BMD | 8.52E-01 |
| Chr8 116096495-119685457 | UK Biobank heel QUS BMD | 1.00E+00 |
| Chr8 126410917-128659111 | UK Biobank heel QUS BMD | 9.95E-01 |
| Chr11 47006137-49866050 | UK Biobank heel QUS BMD | 9.95E-01 |
| Chr11 47006137-49866050 | GEFOS total body BMD | 9.98E-01 |
| Chr11 58780549-62223771 | UK Biobank heel QUS BMD | 9.98E-01 |
| Chr12 102964986-104848696 | UK Biobank heel QUS BMD | 9.11E-01 |
| Chr14 72889615-76444767 | UK Biobank heel QUS BMD | 1.00E+00 |
| Chr14 72889615-76444767 | GEFOS total body BMD | 9.96E-01 |
| Chr14 94325825-95750867 | UK Biobank heel QUS BMD | 9.56E-01 |
| Chr15 50369096-54508528 | UK Biobank heel QUS BMD | 9.96E-01 |
| Chr15 50369096-54508528 | GEFOS total body BMD | 9.85E-01 |
| Chr16 49007926-52035823 | UK Biobank heel QUS BMD | 1.00E+00 |
| Chr16 49007926-52035823 | GEFOS spine BMD | 9.63E-01 |
| Chr16 49007926-52035823 | GEFOS hip BMD | 9.66E-01 |
| Chr16 53382572-55903774 | UK Biobank heel QUS BMD | 9.90E-01 |
| Chr17 52487512-59312755 | UK Biobank heel QUS BMD | 9.89E-01 |
| Chr18 11905440-14440799 | GEFOS OP fracture | 9.94E-01 |
| Chr18 11905440-14440799 | UK Biobank heel QUS BMD | 9.98E-01 |
| Chr18 11905440-14440799 | GEFOS hip BMD | 8.00E-01 |
| Chr19 44744108-46102697 | UK Biobank heel QUS BMD | 9.97E-01 |
| Chr21 38711704-40482902 | GEFOS OP fracture | 1.00E+00 |
| Chr21 38711704-40482902 | UK Biobank heel QUS BMD | 1.00E+00 |
| Chr21 38711704-40482902 | GEFOS total body BMD | 9.90E-01 |

**Table ST7**. Gene of Interest- Colocalization and gene-set enrichment for OP phenotypes CRP according to Hypothesis 3

| Chromosome and Region | Gene (Symbol) and SNP | Function | OP Phenotype p-value | CRP p-value | Posterior Probability H3 |
| --- | --- | --- | --- | --- | --- |
| GEFOS OP Fracture | | | | | |
| Chr18 11905440-14440799 | *LDLRAD4/*  *FAM210A* (rs4796995) | intergenic | 2.91E-12 | 1.26E-04 | 99.4% |
| Chr18 11905440-14440799 | *FAM210A* (rs4635400) | intronic | 1.52E-12 | 1.53E-04 | 99.4% |
| Chr18 11905440-14440799 | *RNMT* (rs3819131) | intronic | 5.25E-12 | 2.18E-04 | 99.4% |
| UK Biobank Heel QUS BMD | | | | | |
| Chr1 25516845-27401867 | *ZDHHC18* (rs75460349) | intronic | 4.23E-05 | 4.88E-10 | 93.0% |
| Chr1 25516845-27401867 | *C1orf172* (rs79598313) | intronic | 1.16E-04 | 1.65E-09 | 93.0% |
| Chr2 28598777-29217559 | *PPP1CB* (rs3768666) | intronic | 7.04E-09 | 2.04E-04 | 97.1% |
| Chr2 28598777-29217559 | *RP11-713D19.1* (rs7601413) | downstream | 1.04E-08 | 4.25E-04 | 97.1% |
| Chr2 28598777-29217559 | *SPDYA* (rs7597048) | intronic | 7.54E-09 | 5.56E-04 | 97.1% |
| Chr2 28598777-29217559 | *TRMT61B* (rs6728045) | intronic | 8.11E-09 | 4.87E-04 | 97.1% |
| Chr8 7153079-9154694 | *RP11-10A14.5/*  *RP11-10A14.8* (rs2929451) | intergenic | 5.48E-47 | 1.02E-10 | 97.0% |
| Chr8 9154694-9640787 | *RP11-115J16.1* (rs4841133) | ncRNA_exonic | 1.51E-21 | 3.31E-19 | 100% |
| Chr8 9640787-10463197 | *MSRA* (rs6982308) | intronic | 1.45E-33 | 7.41E-11 | 93.4% |
| Chr8 11278998-13491775 | *TRPS1* (rs4876611) | intronic | 9.68E-25 | 8.33E-06 | 85.2% |
| Chr11 47006137-49866050 | *C11orf49* (rs3885274) | intronic | 4.83E-26 | 2.19E-05 | 99.5% |
| Chr14 94325825-95750867 | *SERPINA1* (rs28929474) | exonic, nonsynonymous SNV, exon7 | 2.09E-04 | 5.47E-10 | 95.6% |
| Chr14 94325825-95750867 | *SERPINA2P/*  *SERPINA1* (rs112635299) | intergenic | 4.38E-04 | 2.20E-10 | 95.6% |
| Chr16 49007926-52035823 | *SALL1* (rs1965024) | exonic, nonsynonymous SNV, exon2 | 6.87E-14 | 7.74E-19 | 100% |
| Chr16 53382572-55903774 | *FTO* (rs55872725) | intronic | 1.74E-10 | 1.88E-13 | 99.0% |
| Chr18 11905440-14440799 | *FAM210A* (s12955116) | intronic | 4.76E-55 | 7.67E-05 | 99.8% |
| GEFOS total body BMD | | | | | |
| Chr6 125424383-127540461 | *RPS4XP9/*  *RSPO3* (rs13204965) | intergenic | 1.02E-18 | 3.35E-06 | 98.8% |
| Chr11 47006137-49866050 | *C11orf49* (rs7108427) | intronic | 2.44E-13 | 2.01E-04 | 99.8% |
| Chr14 72889615-76444767 | *SERPINA1* (rs28929474) | exonic, nonsynonymous SNV, exon5 | 3.65E-04 | 5.47E-10 | 99.6% |
| Chr14 72889615-76444767 | *SERPINA2P/*  *SERPINA1* (rs112635299) | intergenic | 4.27E-04 | 2.20E-10 | 99.6% |
| GEFOS hip BMD | | | | | |
| Chr6 125424383-127540461 | *MIR588/*  *RNU6-200P* (rs1155938) | intergenic | 5.79E-05 | 4.15E-09 | 96.1% |
| Chr6 125424383-127540461 | *RNU6-200P* (rs3861458) | upstream | 1.43E-04 | 2.29E-09 | 96.1% |
| Chr6 125424383-127540461 | *RNU6-200P/*  *VIMP1* (rs9372842) | intergenic | 9.19E-05 | 2.66E-09 | 96.1% |
| Chr6 125424383-127540461 | *VIMP1* (rs9482757) | ncRNA_exonic | 5.69E-05 | 2.29E-09 | 96.1% |
| Chr6 125424383-127540461 | *VIMP1/*  *PRELID1P1* (rs4339479) | intergenic | 8.77E-05 | 1.70E-09 | 96.1% |
| Chr6 125424383-127540461 | *PRELID1P1* (rs4626447) | ncRNA_exonic | 1.18E-04 | 4.81E-09 | 96.1% |
| Chr6 125424383-127540461 | *PRELID1P1/*  *RPS4XP9* (rs6925607) | intergenic | 3.86E-05 | 3.06E-09 | 96.1% |
| Chr6 125424383-127540461 | *RPS4XP9* (rs853961) | upstream | 8.22E-05 | 4.81E-09 | 96.1% |
| Chr6 125424383-127540461 | *RPS4XP9/*  *RSPO3* (rs853986) | intergenic | 5.95E-05 | 5.57E-09 | 96.1% |
| Chr18 11905440-14440799 | *RP11-973H7.1* (rs2847262) | ncRNA_exonic | 1.47E-04 | 9.64E-08 | 80.0% |
| Chr18 11905440-14440799 | *RP11-973H7.1/*  *PTPN2* (rs11876290) | intergenic | 5.85E-04 | 3.95E-09 | 80.0% |

Legend to Table ST7: Results of common colocalizing genetic variants and their respective genes seen between OP phenotypes and CRP as determined by high posterior probability of H3 arising from distinct causal SNPs. All other posterior probabilities (PP.H0, PP.H1, PP.H2, and PP.H4) were less than 10%.

**Table ST8**

1. Colocalization Results between RA and CRP with a high posterior probability of distinct causal SNPs (PP.H3) of at least 50%.

| Genomic Region (CHR & BP) | Phenotype in colocalization with CRP | PP.H0.abf | PP.H1.abf | PP.H2.abf | PP.H3.abf | PP.H4.abf | PP abf distinct causal  variant |
| --- | --- | --- | --- | --- | --- | --- | --- |
| Chr 6, 30798168-31571218 | UK Biobank RA | 9.46E-15 | 1.33E-13 | 6.73E-02 | **9.31E-01** | 1.89E-03 | 93.1% |
| Chr 6  31571218-32682664 | UK Biobank RA | 4.76E-30 | 6.15E-127 | 5.34E-04 | **6.92E-01** | 3.08E-01 | 69.2% |

1. Gene Enrichment Results between RA and CRP with a high posterior probability of distinct causal SNPs (PP.H3) of at least 50%.

| Chromosome and Region | Gene (Symbol) and SNP for RA | Gene (Symbol) and SNP for CRP | Posterior Probability H3 |
| --- | --- | --- | --- |
| Chr 6, 30798168-31571218 | *HCP5/MICB* (rs9267325) intergenic  p-value: 9.40E-18 | *HCP5/MICB* (rs9267325) intergenic  p-value: 7.57E-05 | 93.1% |
| Chr 6  31571218-32682664 | *HLA-DRB1/HLA-DQA1* (rs2647062) intergenic  p-value: 9.94E-130 | *HLA-DRB1/HLA-DQA1* (rs2647062) intergenic  p-value: 8.51E-10 | 69.2% |

**Gene Ontology**

**Table ST9.** Classic results of gene ontology enrichment analysis results for RA, CRP and OP variables for GO term *GO:0005515.*

| Variables | Rank (Node) | Annotated | Significant | Expected | p-value (Fisher test) |
| --- | --- | --- | --- | --- | --- |
| Hip BMD | 3 | 13845 | 10787 | 10600 | 1.20E-10 |
| Spine BMD | 2 | 13842 | 10811 | 10585 | 1.40E-19 |
| Total body BMD | 2 | 13838 | 11587 | 11425 | 2.40E-14 |
| UKB QUS heel BMD | 3 | 13775 | 10268 | 10166 | 3.10E-06 |
| UKB OP self-reported | 1 | 13775 | 10918 | 10726 | 1.40E-15 |
| RA | 2 | 13590 | 8382 | 8162 | 3.30E-10 |
| CRP | 2 | 13851 | 9564 | 9406 | 5.90E-07 |

The gene ontology class assessed was ‘molecular functions’ and *GO:0005515* denotes protein binding.

Arm BMD and OP fracture variables of the GEFOS consortium did not reveal any association with the GO term examined, and are therefore not shown here.
